# Draft Genome Sequence of *Rhizobium rhizogenes* Strain ATCC 15834

**DOI:** 10.1128/genomeA.01108-14

**Published:** 2014-10-30

**Authors:** Kaisa Kajala, David A. Coil, Siobhan M. Brady

**Affiliations:** a Department of Plant Biology and Genome Center, University of California Davis, Davis, California, USA; b Genome Center, University of California Davis, Davis, California, USA

## Abstract

Here, we present the draft genome of *Rhizobium rhizogenes* strain ATCC 15834. The genome contains 7,070,307 bp in 43 scaffolds. *R. rhizogenes*, also known as *Agrobacterium rhizogenes*, is a plant pathogen that causes hairy root disease. This hairy root induction has been used in biotechnology for the generation of transgenic root cultures.

## GENOME ANNOUNCEMENT

*Rhizobium rhizogenes*, also known as *Agrobacterium rhizogenes* (1), is a Gram-negative soil bacteria and a plant pathogen. *R. rhizogenes* infects a wide range of flowering plants with hairy root disease by introducing root-inducing (Ri) transfer-DNA (T-DNA) into the plant genome (2). The Ri T-DNA expression in plants leads to increased biosynthesis of the plant hormone auxin (3), which affects root growth and promotes lateral root initiation (4). This ability to induce transgenic infinitely growing root systems has been applied for biotechnology and research uses, including production of phytochemicals and recombinant proteins (5) and studies of root development (6–8).

*Rhizobium rhizogenes* strain ATCC 15834 was obtained from ATCC for hairy root culture work with tomato (6). The bacteria was streaked on solid MG/L medium and grown for 2 days at 28°C (7). Cultures of the strain were grown on liquid MG/L for preparation of electrocompetent cells (6) and DNA extraction. Genomic and plasmid DNA was extracted using a PowerSoil DNA Isolation Kit (MO Bio Laboratories, Inc.) from 100 mL fresh overnight culture. Illumina paired-end libraries were then made from sonicated DNA using a TruSeq DNA Sample Prep version 2 kit (Illumina). Fragments between 300 and 600 bp were selected using a Pippin Prep DNA size selection system (Sage Science).

A total of 9,998,182 paired-end reads were generated on an Illumina MiSeq, at a read length of 300 bp. Quality trimming and error correction of the reads resulted in 8,577,816 high-quality reads. All sequence processing and assembly was performed using the A5 assembly pipeline (9). This pipeline automates the processes of data cleaning, error correction, contig assembly, scaffolding, and quality control. The assembly produced 54 contigs contained in 43 scaffolds (minimum, 621 bp; maximum, 1,448,967 bp; *N*_50_, 1,468,096 bp). During scaffolding some contigs were merged based on short overlaps and read-pair information, yielding a final collection of 43 contigs that were submitted to GenBank. This resulted in a final assembly of 7,070,307 bp with a GC content of 60% and an overall coverage estimate of approximately 400×. Completeness of the genome was assessed using the PhyloSift software (10), which searches for a list of 40 highly conserved, single-copy marker genes (11), of which all 40 were found in this assembly.

Automated annotation was performed using the RAST annotation server (12). *R. rhizogenes* strain ATCC 15834 contains 6,919 predicted coding sequences and 54 predicted RNAs. A full-length (1,416 bp) 16S sequence was obtained from this annotation and was used to confirm the *Rhizobium* species by comparison to 54 publicly availably *Agrobacterium* 16S sequences by MUSCLE alignment (13), which was used to construct a phylogenetic tree with FastTree 2 (14).

*R. rhizogenes* ATCC 15834 has been used to induce hairy root cultures in many biotechnology and research applications (5, 6) and its genome sequence will provide a resource for further studies of how the hairy root disease is induced.

### Nucleotide sequence accession numbers.

This whole-genome shotgun project has been deposited at DDBJ/EMBL/GenBank under the accession number JFZP00000000. Illumina reads are available at the ENA Short Read Archive under accession number PRJEB7025.
